# Cost-effectiveness and cost-utility analysis of a nurse-led, transitional care model to improve care coordination for patients with cardiovascular diseases: results from the “Cardiolotse” study

**DOI:** 10.1007/s10198-024-01734-7

**Published:** 2024-11-06

**Authors:** Marie Coors, Wiebke Schüttig, Katrin C. Reber, Harald Darius, Alfred Holzgreve, Sebastian Karmann, Anica Stürtz, Rebecca Zöller, Saskia Kropp, Petra Riesner, Leonie Sundmacher

**Affiliations:** 1 Chair of Health Economics, TUM School of Medicine and Health, Technical University of Munich, Georg-Brauchle-Ring 60/62, 80992 Munich, Germany; 2 AOK Nordost–Die Gesundheitskasse, Health Services Management, Berlin, Germany; 3 Vivantes–Netzwerk für Gesundheit GmbH, Berlin, Germany; 4 Munich Center for Health Economics and Policy, Munich, Germany

**Keywords:** Care coordination, Transitional care, Cardiovascular disease, Economic evaluation, Cost-effectiveness, Health-related quality of life, I19

## Abstract

**Objective:**

To assess the 12-month cost-effectiveness of the nurse-led transitional care program “Cardiolotse” (CL) for patients with cardiovascular diseases compared to usual care (UC).

**Methods:**

A cost-effectiveness analysis (CEA) and cost-utility analysis (CUA) were conducted from the perspective of statutory health insurance (SHI), covering a time horizon of 12 months. Analyzed outcomes included the number of rehospitalizations and health-related quality of life (HRQoL). Total costs comprised program costs and the utilization of healthcare resources. Point estimates are presented as incremental cost-effectiveness ratios (ICERs) and incremental cost-utility ratios (ICURs). Sensitivity and subgroup analyses were conducted to illustrate uncertainty and provide insights into the impact mechanisms of the CL program.

**Results:**

The study population consisted of 2550 patients, with 1256 allocated to the intervention group and 1294 to the control group. Patients who received support from CLs experienced fewer rehospitalizations and lower inpatient costs from an SHI perspective, compared to the UC group. HRQoL assessments indicated higher utility values for CL patients at the 12-month follow-up. Total program costs amounted to €1454.65 per patient. The CEA and CUA demonstrate that the CL program is dominant compared to UC from the SHI perspective.

**Conclusion:**

Our study shows that the CL program not only reduces the number of rehospitalizations and costs but increases HRQoL, resulting in a dominant ICER and ICUR. Further research is necessary to evaluate longer periods of time, different levels of care intensity, and perspectives of different healthcare stakeholders.

**Trial registration:**

German Clinical Trial Register DRKS00020424, 2020-06-18, retrospectively registered.

**Supplementary Information:**

The online version contains supplementary material available at 10.1007/s10198-024-01734-7.

## Introduction

Cardiovascular diseases (CVDs) are linked to high rates of disability, comorbidity, and hospitalization and are the leading cause of death worldwide [1, 2]. The Global Burden of Disease Study reported 523 million cases of CVD in 2019 [3], with their prevalence expected to rise due to demographic and epidemiological changes associated with an aging population [4–6]. CVDs are thus of high public health relevance and a major cost factor for healthcare systems. In Germany, the estimated healthcare costs attributed to CVDs amounted to €56.7 billion in 2020, representing 13.1% of the country’s total healthcare expenditure that year [7].

An important contributor to the social and economic burden of CVDs is their predominantly chronic nature. Rates of readmission shortly after initial hospitalizations are high, increasing the risk of poor health outcomes [6]. Individual health behaviors, such as medication non-adherence, tobacco use and obesity, are key risk factors for readmission [8, 9], but often go unaddressed following hospital discharge. This underscores the importance of the transition from inpatient to outpatient care, and the opportunity it represents to influence these modifiable risk factors through improved care coordination and patient education.

In Germany, hospitals are legally mandated to provide appropriate discharge services to their patients. In practice, however, the transfer of information from inpatient to ambulatory care providers post-discharge primarily occurs through discharge letters. Typically, patients receive a hard copy of this letter, which is also sent to their general practitioner (GP) or other office-based specialists via standard mail. Patients need to arrange their own ambulatory care appointments, and the letters are often written in technical language, demanding high levels of self-organization and health literacy. This process also frequently leads to delays or loss of information for ambulatory care providers [10]**.** Patients often feel overwhelmed by the complexity of this post-discharge process and the fragmented nature of the German healthcare system, reporting inadequate communication and support [11]. Given the frequent lack of suitable and satisfactory post-discharge assistance in Germany, particularly for chronically ill patients, there is a clear need for innovative intersectoral approaches. Such approaches should guide patients through the care process, promote health education, and encourage adherence to recommended therapy plans [12].

International research indicates that comprehensive discharge management is associated with lower hospital readmission rates, improved follow-up care, and better health outcomes for patients with CVDs [13–18]. Furthermore, there is evidence that integrating nurse-led transitional care services in the discharge process can reduce all-cause and cause-specific hospitalizations, all-cause mortality, and the length of hospital stay [19, 20]. Given these findings, it can be expected that effective discharge services, particularly those involving nurse-led transitional care, have the potential not only to decrease the medical and psychological burden on patients and their relatives, but also to ease the economic and financial burden on healthcare systems. Indeed, in the case of nurse-led disease management programs for patients with heart failure, a systematic review conducted by Bierman et al. suggests savings of up to €7,330 per rehospitalization prevented [21]. To our knowledge, however, no study to date has investigated the cost-effectiveness of nurse-led, post-discharge transitional care programs for patients with other CVDs, particularly in the German healthcare setting.

The aim of the present study was to fill this gap in the literature by conducting a cost-effectiveness and a cost-utility analysis of the innovative care program “Cardiolotse” (CL; German for “Cardiac care navigator”). Designed to improve the transition from the inpatient to outpatient sector in Germany, the program offered individualized support for patients with chronic CVDs. Its effectiveness was evaluated through a randomized controlled trial (RCT). The key component of the program was the CL, a specially trained medical care manager whose primary tasks were to assist patients during the post-discharge process, guiding them through the healthcare system, providing prevention advice, and facilitating access to medical resources. We examined the cost-effectiveness of the CL program compared to usual care (UC) from a statutory health insurance (SHI) perspective 12 months after the start of the intervention.

## Methods

### Setting and intervention

The underlying clinical study was a prospective RCT evaluating the effects of the new healthcare program on patients with coronary heart disease (CHD), cardiac arrhythmia (CA), or heart failure (HF) compared to UC. From January 2019 to March 2020, patients were recruited in the Department of Internal Medicine across eight sites of the Vivantes Hospital Group in Berlin, Germany.

Participants were randomly allocated in a 1:1 ratio to either the treatment (CL) or control group (UC). The recruiting CL was blinded to the allocation sequence of the randomization tool. Once patients were assigned to either of the two study groups, it was no longer feasible to maintain the blinding of patients, healthcare providers, or analysts with regard to the status of the randomization. Patients in the control group received UC according to standard protocols during their hospital stay and following their hospital discharge. These protocols included the provision of a hospital discharge letter with follow-up recommendations as well as services and medication available in the German healthcare setting. Patients randomized to the intervention group, however, received additional assistance from the CL during the transition from inpatient to outpatient care and the post-discharge process. This assistance encompassed the provision of a disease-specific information booklet, tailored prevention and follow-up care advice, as well as regular telephone consultations at a priori defined intervals (first quarter following discharge: once a month; second quarter following discharge: every 6 weeks; third and fourth quarters after discharge: every 3 months) after the hospital stay to ensure coordinated post-discharge care. In addition, patients had the opportunity to contact the CL hotline in case of questions.

To meet the specific needs of patients with CVDs and qualify as a CL, interested medical staff underwent supplementary training in areas such as communication skills, physical and rehabilitative treatment options, care services and care delivery mechanisms, and legal and ethical matters. The duration of the training ranged from 160 to 240 h depending on existing qualifications. The staff eligible for this training were either nurses or medical or healthcare assistants working on nurse-led teams. To further improve communication and coordination in outpatient care, regional GPs and cardiologists were informed about the project and invited to formally participate in the program through a contractual agreement.

The intervention period lasted at least 12 months for the entire study population and up to 24 months for a subgroup of participants, depending on their time of recruitment. It was administered consistently, regardless of subsequent hospitalizations that occurred during the follow-up period. The primary endpoint of the RCT was the 12-month all-cause rehospitalization rate, measured as the share of patients with at least one rehospitalization and the average number of rehospitalizations per patient during the 12-month follow-up. Secondary endpoints of the RCT were the indication-specific (CHD, CA, or HF) rehospitalization rate, the length of hospital stay upon rehospitalization, mortality, health-related quality of life (HRQoL), and patient satisfaction. The study protocol, which includes more detailed information on the clinical study design and the scale and scope of the intervention, has been published elsewhere [22].

### Study sample

Using the hospital information system (HIS) of the Vivantes Hospital Group, the CLs systematically identified eligible patients for the study prior to their hospital discharge by means of predefined inclusion and exclusion criteria. The target population consisted of adult patients (aged 18 or above) with CHD (ICD 10-GM I20-I25), CA (ICD 10-GM I47-I49), or HF (ICD-GM 10 I50). In addition, patients were required to be insured by AOK Nordost, the statutory health insurer with the largest market share in northeast Germany [23]. Exclusion criteria were pre-existing cognitive or severe psychiatric diagnoses (ICD 10-GM F20-F29, F33.3), a recorded long-term care level greater than three (on a scale of five), or residence in a long-term care facility. Eligible patients had to provide written, informed consent to participate in the study. The sample size calculation indicated that a minimum of 2,188 analyzable patients, or 1,094 per study group, was necessary to detect a 20% reduction in the primary endpoint, the 12-month all-cause rehospitalization rate [22].

### Type of economic evaluation

The prospective economic evaluation analyzed the costs and outcomes of CL care compared to UC 12 months after the start of the intervention. The reported point estimates are expressed as incremental cost-effectiveness ratios (ICERs) and incremental cost-utility ratios (ICURs), which quantify the additional costs in euros (€) relative to each additional unit of effectiveness (rehospitalization) and quality-adjusted life year (QALYs), respectively. Both the cost-effectiveness analysis (CEA) and the cost-utility analysis (CUA) were performed from the SHI perspective. The available dataset consisted of administrative claims data (spanning 12 months before and up to 24 months after the index hospitalization) provided by AOK Nordost, along with primary clinical data collected during the trial. Owing to the 12-month follow-up period, discounting of costs and effectiveness measures was not applicable. The design of this economic evaluation was developed in accordance with the established Consolidated Health Economic Evaluation Reporting Standards [24].

#### Health-related outcomes

The effectiveness measure for the CEA was the incremental change in the all-cause rehospitalization rate between the intervention and control groups. In alignment with the primary effectiveness outcome, the rehospitalization rate was defined as the average number of all-cause rehospitalizations within 12 months and calculated based on the documented and billed hospitalizations from the administrative claims dataset. Furthermore, the indication-specific rehospitalization rate was examined as a secondary endpoint. These considered hospitalizations were coded with at least one of the index indications (ICD 10-GM I20-I25, I47-I49, I50) as either the main or secondary diagnosis. To prevent overestimation, the number of rehospitalizations did not include the index hospitalization for study recruitment, any care received in a hospital assessment or observation unit prior to inpatient admission, or any intra-hospital or inter-institution transfers.

For the CUA, HRQoL was measured using the five-level version of the EQ-5D questionnaire (EQ-5D-5L), which consists of five quality-of-life dimensions (mobility, self-care, usual activities, pain/discomfort, and anxiety/depression). Each dimension has five levels of severity (no problems, slight problems, moderate problems, severe problems, and extreme problems) [25]. EQ-5D data were collected via telephone interviews at baseline, 3 months, and 12 months after the index hospitalization. Applying the German value set for the EQ-5D-5L [26], the self-reported patient ratings for each dimension were combined to form an average utility score, which ranged from -0.661 to 1.0, with 1 representing perfect health and 0 representing death. Utility scores below zero indicate that patients perceive the health state to be worse than being dead. The EQ-5D-5L values recorded at baseline, 3 months, and 12 months were used to calculate QALYs using the area-under-the-curve method [27].

In addition to the effectiveness and utility measures used for the economic evaluation, the 12-month mortality rate was analyzed to identify any potential systematic bias during the observation period.

#### Costs

The economic evaluation for both the CEA and CUA took into account two primary cost factors from the SHI perspective: program costs and healthcare resource utilization.

Program costs comprised all costs related to the new model of care and were calculated based on trial records and funding documentation. To ensure a detailed assessment, the analysis distinguished between the preparation phase (pre-trial, phase 1) and the intervention phase (during the trial, phase 2), with total program costs being the aggregate of phases 1 and 2. The preparation phase encompassed costs associated with planning and executing the training program for the CLs, including communication training and setting up the necessary IT infrastructure. Intervention phase costs encompassed the personnel costs for the CLs, medical and nursing leadership teams, and ongoing IT system support. Additional costs included payments to participating outpatient care practices for treating patients randomized to the intervention group. Costs related to the administration and evaluation of the trial itself were excluded from the calculation of total program costs. The average program costs over 12 months were calculated by distributing the combined preparation and intervention costs proportionally across patients in the intervention group.

Costs related to the utilization of healthcare resources were calculated based on the administrative claims data provided by the participating SHI provider (AOK Nordost). These included data on the utilization of inpatient and outpatient care, pharmaceuticals, medical aids and therapeutic services, home nursing care and household help, rehabilitation services, sick pay, and medical transport. Sick pay, compensated in Germany by statutory health insurance for work absences beyond six consecutive weeks, was considered only for employed patients.

The costs for inpatient care were calculated excluding the costs accrued during the index hospitalization. Cost components with utilization rates below 20% were combined in a single category labeled “other care services” and included the costs of home nursing care and household help, medical aids and therapeutic services, rehabilitation services, and sick pay. Healthcare resource costs were determined from the SHI perspective by multiplying the unit costs reimbursed by the insurer by the observed utilization rates. Where possible, we additionally identified indication-specific costs via the documented ICD codes.

Lastly, the average total cost per patient per study group was calculated by summing the program costs and the total costs of healthcare resource utilization. If a patient died during the trial, the accumulated costs were considered proportionally up to the day of death. Costs were adjusted for inflation using the Harmonized Index of Consumer Prices of the German Federal Statistics Office, with 2021 being the reference year [28, 29].

#### Statistical analysis

The statistical analysis was performed using an intention-to-treat approach, which included all patients regardless of their adherence to the treatment protocol. Furthermore, the analysis sample was restricted to patients for whom administrative claims data on treatment costs and effectiveness were available. To address systematic non-responder bias, missing EQ-5D-5L data were imputed based on the multiple imputation by chained equations (MICE) method (see Supplement A) as suggested by Faria et al. [30] for within-trial cost-effectiveness analyses.

Before performing the economic evaluation, we compared demographic, socio-economic, and clinical baseline characteristics between the study groups to identify potential imbalances following randomization [31]. Both the primary and secondary outcomes were assessed using univariate and multivariate regression models. The multivariate regression models incorporated the following known and available predictors of rehospitalization in CVD patients as covariates [32–35]: age, gender, and the 12 months pre-trial Charlson comorbidity index (CCI), which measures the risk of death within one year of hospitalization on a scale from zero to 24 [36].

To take account of the nature of the outcome parameters, different regression types were selected based on the assumed underlying distribution of the data: to assess statistical differences in the average number of rehospitalizations between the intervention and control groups, negative binomial regression models were estimated. For HRQoL, differences were assessed using linear regression, controlling for baseline EQ-5D-5L values following the recommendation by Manca et al. [37]. Cost analyses were performed for each cost category separately, as well as for the average total costs, using a gamma-distributed, generalized linear model (GLM) to account for the non-negative, right-skewed distribution of the data. Estimates generated by GLMs are reported as average marginal effects.

In the CEA and CUA, the ICERs and ICURs were calculated by dividing the incremental costs by the incremental effects or utilities. To reflect the correlation between the effect and cost measures instead of assuming their independence, seemingly unrelated regression models (SUR) were used to generate the ICER and ICUR estimates [38]. The reported results of the CEA and CUA represent the incremental costs per rehospitalization avoided and the incremental costs per QALY gained, respectively.

#### Sensitivity and subgroup analyses

The statistical uncertainty around the ICER and ICUR was estimated using the non-parametric bootstrap method with 1000 replications. To graphically represent the distribution of the point estimates, 95% confidence intervals were generated and plotted on a cost-effectiveness plane. In addition, we constructed cost-effectiveness acceptability curves (CEACs) to visualize the probability of accepting the new model of care compared to UC in terms of various willingness-to-pay thresholds.

To investigate the possibly heterogeneous impact mechanisms of the CL program, several subgroup analyses were performed following guidance from medical experts. Subgroups were defined based on the index indications (CHD, CA, HF) and the length of the follow-up period (24-month follow-up was only available for a subset of the participants). To explore the influence of the COVID-19 pandemic, a separate analysis was conducted of the subgroup of patients for whom the 12-month follow-up had been concluded before the onset of the pandemic in mid-March 2020. Furthermore, following the example of Hernández et al. [39] and Ulrich et al. [40], participants were excluded whose costs were above the 95th percentile in order to determine their impact on the cost-effectiveness of the trial.

All findings were interpreted in terms of their clinical relevance and statistical significance, with a significance level set at 5% using two-sided tests. The analyses were conducted using Stata 16.1.

## Results

### Study population

During the recruitment period, a total of 2862 patients were randomized equally into the intervention and control groups. The final study population consisted of 2550 patients due to factors affecting the availability of the administrative claims data, such as data protection reasons or changes in insurance providers, as well as patients exercising their right to withdraw within two weeks of recruitment. Of these patients, 1256 were in the intervention group and 1294 were in the control group. Comparative analysis of baseline characteristics revealed no statistically significant differences between the groups at a 5% significance level (see Table 1).

**Table 1:** Characteristics of the Study Population

Parameter	Mean CL^a^ (Intervention group)	Mean UC^a^ (Control group)	Difference in means^b^
Socio-demographic characteristics			
Mean age	73.48 (12.90)	73.54 (12.42)	0.06 [−0.92, 1.04]
Gender (% female)	47% (n_f_ = 592)	44% (n_f_ = 571)	−0.03 [−0.07, 0.01]
School education^c^, % (n)			
No school leaving certificate	5% (n = 61)	5% (n = 65)	0.00 [−0.02, 0.02]
Basic secondary education	49% (n = 617)	49% (n = 632)	0.00 [−0.04, 0.04]
Intermediate secondary education	22% (n = 282)	20% (n = 261)	−0.02 [−0.05, 0.01]
Vocational/technical secondary education	2% (n = 21)	1% (n = 12)	−0.01 [−0.02, 0.00]
Secondary education qualifying for university admission	5% (n = 68)	5% (n = 63)	−0.01 [−0.02, 0.01]
Professional qualification^d^, % (n)			
No formal training	15% (n = 190)	15% (n = 190)	0.00 [−0.03, 0.02]
Apprenticeship	60% (n = 757)	57% (n = 740)	−0.03 [−0.07, 0.01]
Vocational school	2% (n = 25)	2% (n = 21)	0.00 [−0.01, 0.01]
Technical school	1% (n = 11)	0% (n = 3)	−0.01* [−0.01, 0.00]
Technical college/University of applied sciences	1% (n = 9)	1% (n = 10)	0.00 [−0.01, 0.01]
University	5% (n = 57)	5% (n = 64)	0.00 [−0.01, 0.02]
Clinical characteristics			
Patients with CHD^e^	65% (n = 818)	65% (n = 847)	0.00 [−0.03, 0.04]
Patients with HF^e^	46% (n = 583)	45% (n = 588)	−0.01 [−0.05, 0.03]
Patients with CA^e^	55% (n = 689)	53% (n = 690)	−0.02 [−0.05, 0.02]
Pre-trial CCI	3.23 (2.69)	3.38 (2.80)	0.15 [−0.06, 0.36]
Pre-trial healthcare costs^f^ [€]	10,924.05 (17,889.12)	11,032.12 (14,597.66)	108.30 [−1157.31, 1373.91]
N	1256	1294	2550

^a^ Means/percentage with standard deviation/number of patients in parentheses

^b^ Significance was tested using student t-test or test of proportions, 95% confidence interval in square brackets

^c^Educational attainment according to the German schooling system: 1 Basic secondary education (Volks-, Hauptschule), 2 Intermediate secondary education (Mittlere Reife, Realschule), 3 Vocational/technical secondary education (Fachoberschule), 4 Secondary education qualifying for university admission (Abitur/ EOS), 0 No school leaving certificate

^d^Vocational qualification according to the German professional training education system: 1 Apprenticeship (Lehre), 2 Vocational school (Berusfachschule), 3 Technical school (Fachschule), 4 Technical college/university of applied sciences (Fachhochschule), 5 University (Universität), 0 no training

^e^ Patients may have received multiple diagnoses during their index hospitalization

^f^ Within 12 months prior to the start of the intervention

*** p < 0.01, ** p < 0.05, * p < 0.1

*CA* cardiac arrhythmia, *CCI* Charlson comorbidity index, *CHD* coronary heart disease, *CL* “Cardiolotse” program, *HF* heart failure, *UC* usual care

During the index hospitalization, at least one of the predefined inclusion indications was documented for all patients: 65% had CHD, 46% had HF, and 54% had CA. Patients were 73.5 years of age on average, most had attended at least elementary school, and a majority had completed an apprenticeship. Pre-trial clinical characteristics showed a mean CCI of 3.3, and mean healthcare costs in the 12 months before the index hospitalization were approximately €11,000.

### Clinical outcomes

Table 2 summarizes the main findings of the outcome analyses. Over the 12-month follow-up period, CL patients experienced fewer rehospitalizations on average than UC patients. While the difference was statistically non-significant for all-cause rehospitalizations, we observed a statistically significant reduction in the number of indication-specific rehospitalizations in both the univariate (−0.14; p = 0.03; 95% CI [−0.27, −0.02]) and multivariate (−0.13; p = 0.05; 95% CI [−0.25, 0.00]) regression models. The results for the primary outcome measure of the underlying RCT, which considered the share of patients with at least one rehospitalization, support these findings: 63% of CL patients versus 66% of UC patients were readmitted within the first 12 months (−0.04; p = 0.04; 95% CI [−0.08, 0.00]) (see Supplement B, Table SB1).

**Table 2:** Number of rehospitalizations, health-related quality of life, and mortality over the 12 months after index hospitalization, by group

Parameter	Mean CL^a^	MeanUC^a^	Univariate regression model^b^	Multivariate regression model^b^^c^
# of rehospitalizations				
All causes	1.44 (1.73)	1.55 (1.87)	−0.11 [−0.25, 0.03]	−0.09 [−0.23, 0.05]
Indication-specific	1.23 (1.54)	1.37 (1.72)	−0.14** [−0.27, −0.02]	−0.13** [−0.25, 0.00]
Mortality rate				
12 months (%, n)	15.13% (n = 190)	15.69% (n = 203)	−0.01 [−0.03, 0.02]	0.00 [−0.03, 0.03]
Health-related quality of life^d^ (multiple imputation model)				
Baseline	0.743 (0.300)	0.750 (0.299)	−0.006 [−0.031, 0.019]	−0.006 [−0.030, 0.018]
3 months	0.638 (0.353)	0.629 (0.355)	0.012 [−0.016, 0.041]	0.009 [−0.018, 0.037]
12 months	0.600 (0.381)	0.573 (0.380)	0.029* [−0.003, 0.060]	0.023 [−0.006, 0.053]
QALY^e^ (multiple imputation model)				
12 months	0.637 (0.306)	0.623 (0.302)	0.017 [−0.005, 0.039]	0.013 [−0.007, 0.034]
N	1256	1294	2550	2550

^a^ Means with standard deviation in parentheses

^b^ 95% CI in square brackets

^c^ Adjusted for age, gender, and pre-trial Charlson comorbidity index; only main coefficient displayed

^d^ Quality of life measured by the EuroQol EQ-5D-5L questionnaire with a range from -0.661 to 1.0

^e^ QALYs were estimated using linear regression models based on the EQ-5D-5L values measured at baseline, 3 months, and 12 months

*** p < 0.01, ** p < 0.05, * p < 0.1

*CL* “Cardiolotse” program, *UC* usual care

The mortality rates were similar between the two groups, with no statistically significant difference observed (CL: 15.13% vs UC: 15.69%; p = 0.70).

Regarding HRQoL as measured by the EQ-5D-5L, values were available for 85% of the study population at baseline (CL: 87%, n = 1091; UC: 82%, n = 1,067), for 81% at 3 months (CL: 81%, n = 1016; UC: 81%, n = 1049), and for 63% at 12 months (CL: 61%, n = 768; UC: 65%, n = 841). As previously described, we used a multiple imputation model to handle missing values. HRQoL continuously decreased in both groups during the 12 months after the index hospitalization. Although the difference was not statistically significant at the 5% level, we observed that CL patients reported a higher HRQoL at both 3 months (0.012; p = 0.40; 95% CI [−0.016, 0.041]) and 12 months (0.029; p = 0.08; 95% CI [−0.003, 0.060]) compared to UC patients. On average, CL patients gained an additional 0.017 QALYs over the 12-month intervention period compared to UC patients, although this gain was not statistically significant.

In addition to statistical significance, efforts were made to determine the minimum clinically important difference (MCID) for the HRQoL measurements. MCID represents the smallest change in a health outcome that patients or clinicians perceive as important. Although MCID can vary by country, socio-demographic factors, and disease, a review by Coretti et al. reports typical ranges from 0.03 to 0.54 [41]. Thus, the observed differences in HRQoL after 12 months (0.029; p = 0.08; 95% CI [−0.003, 0.060]), while not statistically significant, could still be relevant from a clinical perspective.

### Resource use and costs

Table 3 presents the main results of the cost analysis. Using administrative claims data, the utilization of healthcare resources across care sectors was examined from the SHI perspective.

**Table 3:** Resource use and costs at 12 months after the index hospitalization, by group

Parameter	Mean CL^a^	Mean UC^a^	Univariate regression model^b^	Multivariate regression model^bc^
Inpatient care				
Utilization	0.69 (0.46)	0.72 (0.45)	−0.03 [−0.06, 0.01]	−0.03 [−0.06, 0.01]
Costs (all causes) [€]	10,491.06 (19,285.54)	12,668.38 (24,005.49)	−2186.83** [−3893.75, −479.90]	−2101.23** [−3777.23, −425.24]
Costs (indication-specific) [€]	9142.67 (18,413.15)	11,272.37 (23,278.54)	−2140.80** [−3789.69, −491.91]	−2161.60*** [−3796.28, −526.92]
Outpatient care				
Utilization	0.96 (0.20)	0.97 (0.18)	−0.01 [−0.02, 0.01]	−0.01 [−0.02, 0.01]
# of GP/cardiologist visits^d^	15.10 (9.86)	15.19 (10.65)	−0.09 [−0.88, 0.71]	−0.06 [−0.84, 0.72]
Costs (all causes) [€]	1170.99 (1042.43)	1220.76 (1099.87)	−49.80 [−133.03, 33.43]	−33.21 [−113.53, 47.10]
Costs (indication-specific) [€]	584.75 (473.33)	587.92 (470.68)	−3.17 [−39.81, 33.47]	−0.54 [−35.92, 34.84]
Pharmaceuticals				
Utilization	0.96 (0.20)	0.97 (0.17)	−0.01 [−0.02, 0.01]	−0.01 [−0.02, 0.00]
Costs [€]	2344.67 (5311.71)	2888.58 (12,637.66)	−546.73 [−1305.23, 211.77]	−396.96 [−959.58, 167.67]
Medical transportation services				
Utilization	0.63 (0.48)	0.65 (0.48)	−0.01 [−0.05, 0.02]	−0.01[−0.05, 0.02]
Costs [€]	731.68 (1646.45)	796.77 (2324.00)	−65.17 [−221.58, 91.24]	−61.75 [−207.62, 84.12]
Other care services				
Utilization				
Utilization home nursing care and household help	0.17 (0.37)	0.20 (0.40)	−0.03* [−0.06, 0.00]	−0.03* [−0.06, 0.00]
Utilization medical aids and therapeutic services	0.13 (0.33)	0.16 (0.37)	−0.03** [−0.06, 0.00]	−0.03** [−0.06, 0.00]
Utilization rehabilitation^e^	0.10 (0.31)	0.11 (0.31)	−0.01 [−0.03, 0.02]	−0.01 [−0.03, 0.02]
Utilization sick pay	0.03 (0.17)	0.03 (0.16)	0.00 [−0.01, 0.01]	0.00 [−0.02, 0.03]
Costs (all causes) [€]	1543.60 (8183.78)	1836.92 (8270.87)	−294.44 [−939.50, 350.62]	−230.94 [−627.20, 165.31]
Costs (indication-specific) [€]	1216.51 (7982.44)	1286.05 (6541.87)	−69.59 [−637.78, 498.60]	−201.16 [−550.31, 148.00]
CL program costs [€]	1454.65	−	−	−
Preparation (phase 1) [€]	121.13	−	−	−
Number of follow-up contacts	8.76 (3.74)	−	−	−
Duration of follow-up contacts [minutes]	20.70 (11.28)	−	−	−
Intervention (phase 2) [€]	1333.52	−	−	−
Total costs [€]				
All causes	17,736.65 (23,388.11)	19,411.41 (31,586.99)	−1677.02 [−3836.72, 482.68]	−1119.21 [−3176.49, 938.08]
Indication-specific	15,474.92 (22,200.12)	16,831.69 (30,299.73)	−1358.40 [−3421.55, 704.74]	−907.17 [−2898.02, 1083.69]
N	1256	1294	2550	2550

^a^ Means with standard deviation in parentheses

^b^ 95% CI in square brackets

^c^ Adjusted for age, gender, and pre-trial Charlson comorbidity index

^d^ Reported # of GP/cardiologist visits represent a minimum due to lump-sum payments in the German healthcare system

^e^ Rehabilitation utilization covered by statutory health insurance; main coefficient displayed

*** p < 0.01, ** p < 0.05, * p < 0.1

*CL* “Cardiolotse” program, *GP* general practitioner, *UC* usual care

Annual costs for inpatient care averaged €10,491 in the intervention group (CL) and €12,668 in the control group (UC). Nearly 88% of the total annual inpatient costs resulted from indication-specific hospitalizations (CL: €9143, UC: €11,272). The results of the univariate (€−2187, p = 0.011; 95% CI [€-3893.75, €-479.90]) and the multivariate (€−2101, p = 0.014; 95% CI [€−3777.23, €−425.24]) regression analyses provide strong evidence of cost savings within the inpatient sector of approximately €2100 per patient per year following the CL intervention.

96% of CL patients and 97% of UC patients received outpatient care and pharmaceuticals. On average, patients visited a GP and/or cardiologist about 15 times within the 12 months following their index hospitalization. The regression analyses did not indicate statistically significant differences between the two groups in terms of the utilization and costs of outpatient care and pharmaceuticals.

Medical transportation services were required for 63% of patients in the CL group and 65% of patients in the UC group. The average annual costs amounted to €732 (CL) and €797 (UC), respectively. Again, there were no significant differences between the intervention and control groups.

Other care services (home nursing care and household help, medical aids and therapeutic services) were utilized to a lesser extent by CL patients. However, there was no significant difference in all-cause or indication-specific costs for these services.

Costs associated with the preparation of the CL program (phase 1) and the active intervention (phase 2) were documented during the trial. Preparation costs, which included conceptualizing the CL qualification program, training the CLs, and providing the necessary IT infrastructure, amounted to €121.13 per CL patient. The average number of contacts between CLs and intervention patients during the follow-up period was approximately nine, with an average duration of 21 min. The requisite personnel costs for the CLs, the medical and nursing care leadership team, and maintaining the IT system added up to €1333.52 per CL patient. Summing the costs for phase 1 and phase 2 yielded total program costs of €1454.65 per CL patient. Additional details regarding the apportionment of the program costs can be found in supplementary material B (Table SB3). It should be noted, however, that costs and investments may vary in other settings and organizations depending on the pre-existing infrastructure.

Lastly, we calculated the total costs for each group by adding up the costs for the utilization of healthcare resources and the program costs (including phase 1 and phase 2), yielding average total costs of €17,736.65 for the CL group and €19,411.41 for the UC group. The estimated incremental cost was €-1677.02 (95% CI [€-3836.72, €482.68]).

### Cost-effectiveness and cost-utility analysis

At 12 months after the index hospitalization, patients who received additional support from the CL program had, on average, 0.11 (95% CI −0.25, 0.03]) fewer all-cause rehospitalizations, a 0.017 (95% CI [−0.005, 0.039]) increase in QALYs, and €1677 (95% CI [€−3836.72, €482.68]) less in accumulated costs from the SHI perspective compared to patients receiving UC. Hence, the results of the base case analysis show that the CL program not only reduces the number of rehospitalizations and costs, but increases HRQoL, resulting in a dominant ICER and ICUR.

### Sensitivity and subgroup analysis

**Fig. 1:**
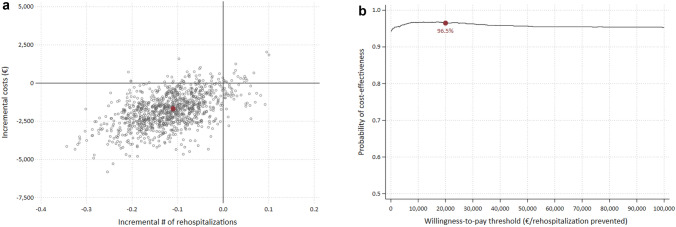
**a** Cost-effectiveness plane for the CEA; red dot indicates base case ICER. **b** Cost-effectiveness acceptability curve (CEAC) for the CEA; red dot indicates the probability of cost-effectiveness at a WTP threshold of 20,000€ per rehospitalization prevented

Figure 1a, b displays the sensitivity analysis for the CEA, including the bootstrapped ICERs in a cost-effectiveness plane and the respective CEAC. Consistent with the point estimate of the base case analysis, a large proportion of the bootstrapped ICERs indicate a reduction in the number of rehospitalizations along with lower accumulated costs in the CL group compared to UC. The probability of the new model of care being cost-effective relative to the willingness-to-pay (WTP) threshold was further visualized in a cost-effectiveness acceptability curve (see Fig. 1b). At a WTP of €0 per rehospitalization prevented, i.e., dominating UC, the probability of the new model of care being cost-effective was estimated at 94.3%.

**Fig. 2:**
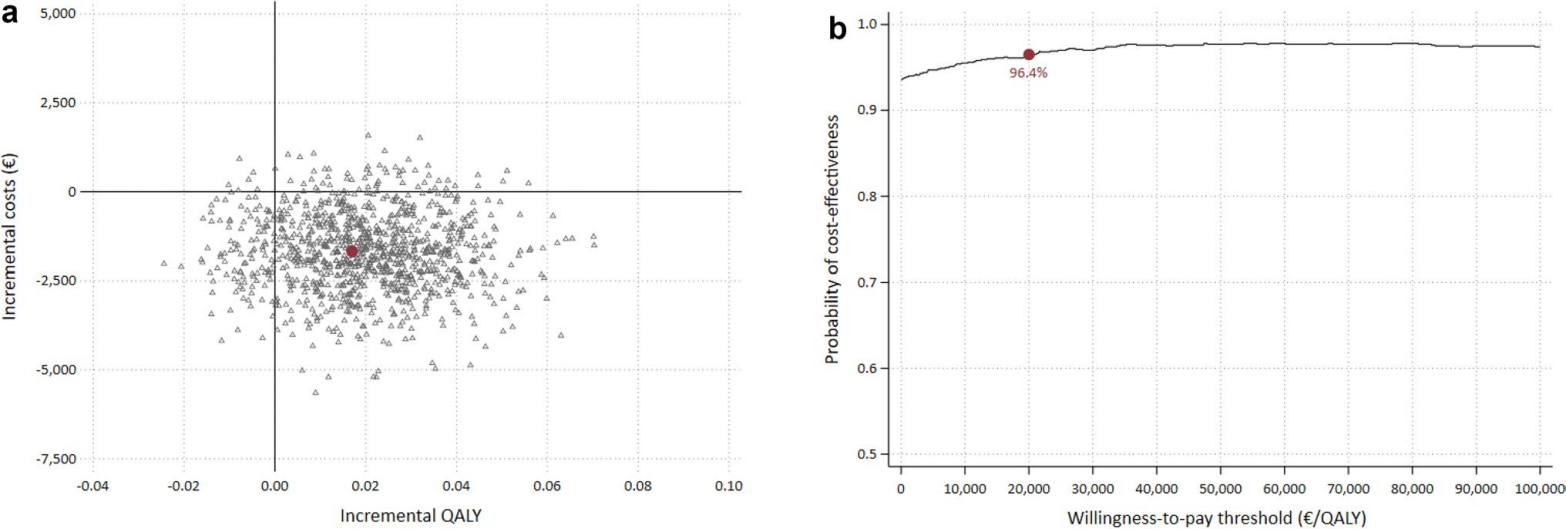
** a** Cost-effectiveness plane for the CUA; red dot indicates base case ICUR. **b** Cost-effectiveness acceptability curve (CEAC) for the CUA; red dot indicates the probability of cost-effectiveness at a WTP threshold of €20,000 per QALY (quality-adjusted life year)

The cost-effectiveness plane for the CUA and the corresponding CEAC are shown in Fig. 2a, b. Again, the bootstrapped results complement the findings of the base case. 93.6% of the estimated ICURs fall in the southeast quadrant, indicating that the CL program was the dominant form of care compared to UC (WTP = 0€ per QALY). While several countries have established cost-effectiveness thresholds for their reimbursement policies [42], Germany does not have an official WTP threshold [43]. A recent empirical study, however, suggests a societal WTP in Germany ranging from €20,000 to €60,000 per QALY, comparable to thresholds in the UK and the Netherlands [44]. The probability of the CL program being cost-effective reached 96.4% at a WTP threshold of €20,000 per QALY and 97.8% at a WTP threshold of €60,000 per QALY.

The detailed results of the subgroup analyses can be found in supplementary material B (Table SB4). A brief summary is as follows: among patients with CHD, who constituted approximately 65% of the study population, the results of the CEA and CUA are consistent with the previously reported main results but reach significance only at the 10% level. However, for patients with CA or HF, no significant differences were observed in terms of the number of rehospitalizations or total costs. Analysis of the subgroup of patients with a 12-month follow-up completed before March 2020 shows a further reduction in the number of rehospitalizations (−0.27, p = 0.09; 95% CI [−0.22, 0.01]) compared to the previously reported main results, which indicates a potential underestimation of the intervention effect due to the consequences of the COVID-19 pandemic. When excluding the 5% of participants who generated the highest costs, the significant difference in the number of indication-specific rehospitalizations persisted, although the magnitude of the difference in total costs decreased to €−279.87 (95% CI [−1321.91, 762.18]). At 24 months after the index hospitalization, no significant difference was observed between patients who received the intervention for 24 months compared to their UC counterparts. For each subgroup, the estimated ICERs support the base case findings that the CL program was the dominant form of care in comparison to UC.

## Discussion

CVDs account for over one-third of global deaths and are responsible for a significant share of total healthcare expenditure in high-income countries [2, 7, 45]. This highlights the need for effective and efficient management options. A key contributor to high rehospitalization rates is the loss of information during the transition from inpatient to outpatient care, resulting in poor care coordination and inadequate treatment. The “Cardiolotse” program was introduced to improve this transition by ensuring continuous and complete information exchange throughout the post-discharge process, supplementing existing discharge services. CLs are trained medical care managers, who support patients with chronic CVDs by assisting them in navigating the healthcare system and accessing medical resources after their hospital stay. The aim of the present study was to examine the cost-effectiveness of the program from the SHI perspective 12 months after the start of the intervention compared to UC.

The analysis of the clinical outcomes showed a significant reduction in the number of indication-specific rehospitalizations and the share of patients with at least one rehospitalization, which significantly reduced mean healthcare costs for inpatient care. In terms of HRQoL, we found that patients cared for by the CL reported a higher utility value at the 12-month follow-up compared to UC patients. Based on the analysis of clinical outcomes and costs, the CEA and CUA demonstrated that the “Cardiolotse” program was the dominant form of care compared to UC from the SHI perspective.

Our results contribute to the existing but limited body of research on the cost-effectiveness of nurse-led transitional care programs for patients with CVDs. In a systematic review of nurse-led disease management programs (DMPs) for patients with HF, Biermann et al. reported that more than half of the included studies showed a statistically significant decrease in the number of all-cause rehospitalizations. Further calculations revealed ICERs ranging from €490 up to €7330 saved per rehospitalization prevented [21]. Given that the authors restricted their search to patients with HF, our analysis extends the findings to patients with CHD and CA.

Similar to the CL program, the Interdisciplinary Network for Heart Failure (HNC) study implemented a structured collaborative disease management program, including in-hospital face-to-face contact between specialist nurses and patients and telephone consultations after hospital discharge [46]. In contrast to our findings, Angermann et al. report higher readmission rates along with a lower mortality risk compared to usual care [46]. From an economic perspective, the cost-effectiveness and cost-utility ratio of the HNC program compared to usual care was estimated to be 8284 € per death avoided and 49,335 € per QALY gained [47]. In addition to the restriction to HF patients and differences in the scope and curriculum of the nurse training program, we identified the follow-up period of six months compared with one year for the CL program as another key difference. Although the one-year observation period cannot fill the gap for evaluating long-term effects, it does suggest that the extended period of CL support may be a key driver for the observed reduction in the number of rehospitalizations.

Also focusing on patients with chronic HF, Herold et al. evaluated a telemonitoring program in Germany led by specially trained nurses. 12 months after the intervention, the authors observed a reduction in average total healthcare costs by €1104 in rural regions and €72 in urban regions [48]. The comparatively smaller reduction in healthcare costs compared to the CL program (€−2101, p = 0.014; 95% CI [€−3777.23, €−425.24]) may be attributed to the difference in upfront investments. A key component of the CL program was the direct, in-person interaction between the CL and the patient during the index hospitalization, contributing substantial personnel costs to the overall program costs of approximately €1,300 per patient. However, such face-to-face engagement likely fosters stronger trust relationships and reduces communication barriers compared to telemonitoring approaches. As a result, it is expected that patients develop greater health literacy and improved adherence to their treatment plans.

In 2022, Driscoll et al. published the results of an integrated review examining the clinical effectiveness and cost-effectiveness of nurse-led, post-discharge services for patients admitted with acute HF [49]. All eight studies included in the review found the intervention to be cost-effective compared to UC. Reported ICERs ranged from $18,259 (Canadian dollars) per life year gained to €40,321 per QALY gained, underscoring the potential of nurse-led, post-discharge services in diverse international healthcare settings.

To our knowledge, there are no other interventions within the framework of a large-scale, representative RCT that are comparable to the “Cardiolotse” program in terms of its scale and scope. While many studies have focused on specific CVD patient subgroups, particularly those with HF, the CL program was designed for a more diverse population comprising patients with CHD, CA, and HF. Furthermore, the detailed breakdown of program costs, categorized into preparation and intervention phases, offers new insights for potential reimbursement strategies to be considered by health insurance providers and policymakers.

Several limitations should be considered when interpreting the results of our study. Because the economic evaluation followed a trial-based design, the intervention period was limited to 12 months of pre-determined care intensity. While our subgroup analyses addressed the impact of 12 versus 24 months of CL support, the study was powered for the primary endpoint, not secondary endpoints or subgroups. Thus, further research is necessary to evaluate the cost-effectiveness over a longer period and with varying levels of care intensity, such as different frequencies of CL-patient interactions.

The within-trial economic evaluation benefitted from the comprehensiveness of the available outcome and cost data. Costs related to the utilization of healthcare resources were estimated based on administrative claims data from a large statutory health insurer, encompassing both actual quantities and reimbursed prices. Nevertheless, average patient characteristics may differ between statutory health insurers, limiting the generalizability of our results.

The HRQoL data were collected during the trial and based on patient reports, rendering them susceptible to recall bias and missing data. To address these concerns, we used the validated EQ-5D-5L questionnaire and a multiple imputation method, respectively. We observed a reduction in patient participation in follow-up questionnaires along with a decrease in the self-reported HRQoL. While patient drop-out was anticipated due to mortality and the potential patient-perceived burden of the trial, the consistent decline in reported quality of life was unexpected. This might be attributable to three factors: first, for the majority of participants, the intervention period coincided with the COVID-19 pandemic, which was associated with significantly decreased HRQoL, increased mental health issues, and heightened anxiety levels [50]. Secondly, participants were recruited for the trial during their first hospitalization following a cardiovascular diagnosis of CHD, CA, or HF. This period marks a mentally and physically challenging experience for patients and often requires intensive subsequent care. Furthermore, the observed CVDs are chronic in nature and progressive, which may also result in a decline in perceived HRQoL over time. Thirdly, there is a discernable increase between the pre-trial CCI (CL: 3.23; UC: 3.38) and the post-trial CCI (CL: 4.77; UC: 4.90). Although no significant differences in the CCI were detected between the groups, the accumulation of comorbidities may exert an influence on the patient-perceived HRQoL.

While statutory health insurers in Germany cover most healthcare costs, our study’s perspective does not account for costs borne directly or indirectly by patients or their families, such as over-the-counter medications, lost productivity, and co-payments. Despite this limitation, the findings from the SHI perspective offer a practical and actionable basis for healthcare professionals and policymakers when making decisions about resource allocation.

If we extrapolate our results to the national level, implementing the CL program has the potential to reduce inpatient treatment costs for CVD patients from the SHI perspective. In 2022, approximately 1.25 million cases of CHD, CA, or HF as the main diagnosis were treated in German hospitals and covered by SHI providers [51]. Assuming the characteristics of our study population are representative (see Supplement C), nationwide implementation of the CL program could prevent up to 54,000 rehospitalizations and save SHI providers a total of €379 million annually. However, from a meso-economic perspective, the cost savings for SHIs could lead to proportional revenue losses for hospitals due to the segmentation and reimbursement structure in the German healthcare system. Furthermore, introducing a new role like the CL might prompt shifts in personnel distribution, potentially exacerbating the existing shortage of skilled healthcare workers. Therefore, it is imperative to consider the diverse incentives and concerns of different healthcare stakeholders in decision-making, with careful attention to financing and reimbursement strategies, employment structures, and the potential redistribution of specialized personnel.

## Conclusion

This study aimed to assess the cost-effectiveness of the nurse-led transitional care program “Cardiolotse” for patients with CVDs. The results indicate that the CL program was associated with a reduction in the number of indication-specific hospitalizations and inpatient care costs. The CEA and CUA demonstrated that CL was the dominant form of care compared to usual care from an SHI perspective 12 months after the start of the intervention. From an economic perspective, the results suggest that nurse-led transitional care programs may be a cost-effective and practical addition to conventional discharge services and post-discharge care for CVD patients. These findings can contribute to the ongoing policy debate on the integration of such programs into healthcare systems. Further research is necessary to evaluate costs and effects over longer periods of time, across different levels of care intensity, and from the perspectives of different healthcare stakeholders.

## Supplementary Information

Below is the link to the electronic supplementary material.

Supplementary file1 (PDF 249 KB)

Supplementary file2 (DOCX 57 KB)

Supplementary file3 (DOCX 47 KB)

## Data Availability

No data are available.
