# Supplementary material for: Cost-effectiveness and cost-utility analysis of a nurse-led, transitional care model to improve care coordination for patients with cardiovascular diseases: results from the “Cardiolotse” study
Source: Eur J Health Econ. 2024 Nov 6;26(5):697–710. doi: 10.1007/s10198-024-01734-7 (PMC12204867; doi:10.1007/s10198-024-01734-7)
Supplement: Supplementary file 1 — Supplementary file1 (PDF 249 KB) [file 10198_2024_1734_MOESM1_ESM.pdf]

Supplement A – Multiple imputation model for HRQoL data at baseline, 3 months, and 12 months

Step 1: For patients who died during the intervention period and therefore could not be interviewed at respective time points, a health-related quality of life score of 0 was assigned. The suffix _*verst* marks variables containing imputed data for deceased patients.

Table SA1: Description of variables relevant for the MI model

Variable	Description	Missing values, %			Range	Mean	SD
		Total	CL	UC			
	Baseline variables						
STUDIENGRUPPE	Study group	0	0	0	0,1	-	-
GESCHLECHT	Female or male	0	0	0	0,1	45.6% female	-
Alter_Einschluss	Age at trial entry	0	0	0	19 to 99	73.512	12.658
charlindex_pre	Pre-trial CCI	0	0	0	0 to 16	3.308	2.748
KHK_PID	CHD patient	0	0	0	0,1	-	-
HRH_PID	CA patient	0	0	0	0,1	-	-
HI_PID	HF patient	0	0	0	0,1	-	-
VERSTORBEN	Patient deceased	0	0	0	0,1	34.1%	-
KH_VWD_ini_PSN	Avg. length of hospital stay (days)	0	0	0	0 to 168	8.016	8.545
	HRQoL variables						
EQ_0M	EQ-5D at baseline	15.92	13.77	18.01	-0.661 to 1.000	0.752	0.293
EQ_0M_verst	EQ-5D at baseline incl. deceased patients	15.37	13.14	17.54	-0.661 to 1.000	0.747	0.298
EQ_3M	EQ-5D at 3 months	26.75	26.83	26.66	-0.661 to 1.000	0.699	0.304
EQ_3M_verst	EQ-5D at 3 months incl. deceased patients	19.02	19.11	18.93	-0.661 to 1.000	0.632	0.355
EQ_12M	EQ-5D at 1year	53.06	54.78	51.39	-0.341 to 1.000	0.737	0.263
EQ_12M_verst	EQ-5D at 1 year incl. deceased patients	36.90	38.85	35.01	-0.341 to 1.000	0.548	0.394

CL indicates "Cardiolotse" program, UC indicates usual care; suffix _verst marks variables containing imputed data for deceased patients.

Step 2: Run multiple imputation model with chained equation using the STATA command *ice*

```
ice EQ_0M_verst EQ_3M_verst EQ_12M_verst ///
STUDIENGRUPPE charlindex_pre GESCHLECHT VERSTORBEN Alter_Einschluss
KHK_PID HI_PID HRH_PID KH_VWD_ini_PSN, ///
saving(HRQoL_MI_aggregated, replace) m(40) match genmiss(ind_miss)
by(STUDIENGRUPPE) seed(10) // predictive mean matching used because
QALYs are non-normally distributed
```

Variable	Command	Prediction equation
STUDIENGR~E		[No missing data in estimation sample]
charlinde~e		[No missing data in estimation sample]
GESCHLECHT		[No missing data in estimation sample]
VERSTORBEN		[No missing data in estimation sample]
Alter_Ein~s		[No missing data in estimation sample]
KHK_PID		[No missing data in estimation sample]
HI_PID		[No missing data in estimation sample]
HRH_PID		[No missing data in estimation sample]
KH_VWD_in~N		[No missing data in estimation sample]
EQ_0M_verst	regress	EQ_3M_verst EQ_12M_verst STUDIENGRUPPE charlindex_pre GESCHLECHT VERSTORBEN Alter_Einschluss KHK_PID HI_PID HRH_PID KH_VWD_ini_PSN
EQ_3M_verst	regress	EQ_0M_verst EQ_12M_verst STUDIENGRUPPE charlindex_pre GESCHLECHT VERSTORBEN Alter_Einschluss KHK_PID HI_PID HRH_PID KH_VWD_ini_PSN
EQ_12M_ve~t	regress	EQ_0M_verst EQ_3M_verst STUDIENGRUPPE charlindex_pre GESCHLECHT VERSTORBEN Alter_Einschluss KHK_PID HI_PID HRH_PID KH_VWD_ini_PSN

Fig. SA1 *Prediction equations from the STATA "ice" command*
