# Supplementary material for: Cost-effectiveness and cost-utility analysis of a nurse-led, transitional care model to improve care coordination for patients with cardiovascular diseases: results from the “Cardiolotse” study
Source: Eur J Health Econ. 2024 Nov 6;26(5):697–710. doi: 10.1007/s10198-024-01734-7 (PMC12204867; doi:10.1007/s10198-024-01734-7)
Supplement: Supplementary file 2 — Supplementary file2 (DOCX 57 KB) [file 10198_2024_1734_MOESM2_ESM.docx]

**Supplement B – Further results**

Table SB1: Share of patients with rehospitalizations within one year of the index hospitalization by group

| **Parameter** | **Mean**  **CL^+^** | **Mean**  **UC^+^** | **Univariate regression model^§^** | **Multivariate**  **regression model^a§^** |
| --- | --- | --- | --- | --- |
| *Share of patients with rehospitalizations* | | | | |
| All causes | 0.63  (0.48) | 0.66  (0.47) | -0.04**  [-0.08, 0.00] | -0.03*  [-0.07, 0.00] |
| Indication-specific | 0.58  (0.49) | 0.61  (0.49) | -0.03*  [-0.07, 0.00] | -0.03  [-0.07, 0.01] |

CL indicates “Cardiolotse” program, UC indicates usual care; ^a^adjusted for age, gender, and pre-trial Charlson Comorbidity Index; only main coefficient displayed; ^§^95% CI in square brackets; ^+^Means with standard deviation in parentheses;

*** p<0.01, ** p<0.05, * p<0.1

*Table SB2: Health-related quality of life within one year of the index hospitalization by group – alternative imputation methods*

| **Parameter** | **Mean**  **CL^a^** | **Mean**  **UC^a^** | **Univariate regression model^b^** | **Multivariate**  **regression model^bc^** |
| --- | --- | --- | --- | --- |
| *Health-related quality of life^d^ (without imputation)* | | | | |
| Baseline | 0.750  (0.293) | 0.755  (0.294) | -0.005  [-0.030, 0.020] | -0.005  [-0.029, 0.019] |
| 3 months | 0.706 | 0.692 | 0.012 | 0.012 |
|  | (0.299) | 0.309 | [-0.016. 0.040] | [-0.015, 0.039] |
| 12 months | 0.753  (0.252) | 0.722  (0.272) | 0.040***  [0.011, 0.070] | 0.039***  [0.011, 0.068] |
| N |  |  |  |  |
| *QALY^e^ (without imputation)* | |  |  |  |
| 12 months | 0.744  (0.222) | 0.725  (0.240) | 0.015***  [0.004, 0.026] | 0.026**  [0.001, 0.051] |
| N |  |  |  |  |
| *Health-related quality of life^d^ (*imputed data for deceased patients*)* | | | | |
| Baseline | 0.744  (0.299) | 0.750  (0.298) | -0.006  [-0.031, 0.019] | -0.006  [-0.030, 0.018] |
| 3 months | 0.639  (0.352) | 0.626  (0.357) | 0.011  [-0.019, 0.042] | 0.011  [-0.019, 0.040] |
| 12 months | 0.557  (0.395) | 0.540  (0.392) | 0.026  [-0.013, 0.065] | 0.023  [-0.013, 0.059] |
| N |  |  |  |  |
| *QALY^e^ (*imputed data for deceased patients*)* | | |  |  |
| 12 months | 0.607  (0.322) | 0.591  (0.322) | 0.020  [-0.012, 0.052] | 0.021  [-0.009, 0.051] |
| N |  |  |  |  |

CL: “Cardiolotse” program; UC: usual care; ^a^means with standard deviation in parentheses; ^b^95% CI in square brackets; ^c^adjusted for age, gender, and pre-trial Charlson Comorbidity Index; only main coefficient displayed; ^d^Quality of life measured by the EuroQol EQ-5D-5L questionnaire with a range from -0.661 to 1.0; ^e^QALYs were estimated using linear regression models based on the EQ-5D-5L values measured at baseline, 3 months, and 12 months;

*** p<0.01, ** p<0.05, * p<0.1

*Table SB3: Apportionment of the CL program costs*

|  | **Total costs (€)** | **Costs per CL patient (€)** |
| --- | --- | --- |
| **CL Program** | 1,827,902.04 | 1,454.65 |
| **Phase 1: Pre-trial preparation** | 152,137.64 | 121.13 |
| CL qualification^1^ | 111,378.94 | 88.68 |
| Planning and execution | 34,511.57 | 27.48 |
| Personnel costs CL | 70,917.37 | 56.46 |
| Communication training | 5,950.00 | 4.74 |
| IT infrastructure^2^ | 19,511.57 | 15.54 |
| Overhead costs^3^ | 21,240.94 | 16.91 |
| **Phase 2: Intervention** | 1,674,902.04 | 1,333.52 |
| Personnel costs | 1,327,440.70 | 1,056.88 |
| CL | 1,021,320.54 | 813.15 |
| Medical leadership team | 159,000.00 | 126.59 |
| Nursing leadership team | 147,120.16 | 117.13 |
| IT system support | 87,946.58 | 70.02 |
| Overhead costs^3^ | 255,566.76 | 203.48 |
| Payments to outpatient care practices | 3,948.00 | 3.14 |

CL: Cardiolotse; ^1^ curriculum elements: communication skills, physical and psychosocial health needs of people with CVDs, physical and rehabilitative treatment options, care services and care delivery mechanisms, and legal and ethical matters; ^2^ equipment and installation of the telephone system, customization of the pre-existing hospital information system; ^3^ Overhead costs are calculated as a proportion of personnel costs and refer to ongoing operating costs such as rent, administration, accounting, human resources, etc.

Table SB4: Multivariate regression model results of the subgroup and sensitivity analyses

|  | **Patients with**  **CHD** | **Patients with**  **CA** | **Patients with**  **HF** | **12-month follow-up prior to COVID** | **Upper 5%**  **excluded** | **24-months**  **follow-up** |
| --- | --- | --- | --- | --- | --- | --- |
| *# of rehospitalizations* |  |  |  |  |  |  |
| All causes | -0.12  [-0.29, 0.05] | -0.08  [-0.27, 0.11] | -0.13  [-0.34, 0.07] | -0.20  [-0.54, 0.14] | -0.08  [-0.21, 0.04] | 0.06  [-0.18, 0.30] |
| Indication-specific | -0.14*  [-0.29, 0.02] | -0.13  [-0.31, 0.04] | -0.14  [-0.34, 0.05] | -0.27*  [-0.59, 0.04] | -0.10*  [-0.22, 0.01] | -0.01  [-0.23, 0.22] |
| *QALY^±^* |  |  |  |  |  |  |
|  | 0.005  [-0.020, 0.030] | 0.006  [-0.023, 0.035] | -0.003  [-0.037, 0.030] | -0.003  [-0.054, 0.049] | 0.013  [-0.008, 0.034] | / |
| *Total costs* |  |  |  |  |  |  |
| All causes | -1,264.71  [-3,826.30, 1,296.89] | -1,959.12  [-4,866.37, 948.13] | -2,662.28  [-5,988.72, 664.16] | -439.83  [-5,746.69, 4,867.03] | -279.87  [-1,321.91, 762.18] | -487.98  [-3,994.27, 3,018.31] |
| Indication-specific | -756.10  [-3,221.55, 1,709.36] | -2,001.13  [-4,847.44, 845.17] | -2,210.33  [-5,445.00, 1,024.34] | -478.02  [-5,635.16, 4,679.11] | -7.35  [-958.93, 944.23] | -460.62  [-3,760.96, 2,839.72] |
| ICER | dominant | dominant | dominant | dominant | dominant | dominant |
| ICUR | dominant | dominant | 810,358.82 | 788,167.22 | dominant | / |
| N | 1,665 | 1,379 | 1,171 | 470 | 2,424 | 1,565 |

CA: cardiac arrhythmia (ICD 10-GM I47-I49); CHD: coronary heart disease (ICD 10-GM I20-I25); HF: heart failure (ICD-GM 10 I50); only main coefficients displayed; regression analyses adjusted for age, gender, and pre-trial Charlson Comorbidity Index; 95% CI in square brackets; *** p<0.01, ** p<0.05, * p
