# Supplementary material for: Cost-effectiveness and cost-utility analysis of a nurse-led, transitional care model to improve care coordination for patients with cardiovascular diseases: results from the “Cardiolotse” study
Source: Eur J Health Econ. 2024 Nov 6;26(5):697–710. doi: 10.1007/s10198-024-01734-7 (PMC12204867; doi:10.1007/s10198-024-01734-7)
Supplement: Supplementary file 3 — Supplementary file3 (DOCX 47 KB) [file 10198_2024_1734_MOESM3_ESM.docx]

**Supplement C – Extrapolation of results to the national level (Germany)**

Table SC1: Number of hospital cases in Germany differentiated by ICD-10 (2021)

| ICD-10 (main diagnosis) | # of hospital cases (adults) |
| --- | --- |
| I20-I25 | 552,605 |
| I47-I49 | 401,268 |
| I50 | 438,257 |
| Total | 1,392,130 |

Source: Statistisches Bundesamt (Destatis), 2023 [51]

Additional information and assumptions:

1. 90% of patients are covered by SHI [22].
2. The average number of hospitalizations for patients with CHD, CA, or HI is independent of the health insurance provider (private vs. statutory health insurance).
3. Initially hospitalized patients with CHD, CA, or HI experience approx. 1.4 indication-specific rehospitalizations within 12-months of the first hospitalization (see Table 2), and 2.4 hospitalizations in total.
4. 20% of hospitalized patients with CHD, CA, or HI are either not eligible for CL care due to pre-existing cognitive or severe psychiatric disorders or living in long-term care facilities, or are not interested in accessing the additional care provided by the CL.
5. The estimated reduction in the number of indication-specific rehospitalizations due to the CL intervention is 0,13 rehospitalizations per patient (see Table 2).
6. The estimated savings in total costs due to the CL intervention are 907.17€ per patient (see Table 3).
7. The preparation and intervention costs for the CL program are covered by SHIs.

Table SC2: Estimated number of annually preventable indication-specific rehospitalizations following a nationwide CL implementation

|  | |  |
| --- | --- | --- |
| # of cases covered by SHI | | 1,252,917 |
| # of hospitalized patients with CHD, CA, or HI | | 522,049 |
| - | # of hospitalized patients not eligible for/not interested in CL care | 104,410 |
| # of patients eligible for CL care | | 417,639 |
| * | Estimated reduction of indication-specific rehospitalization per patient due to the CL implementation | 0.13 |
| # of preventable indication-specific rehospitalizations following a nationwide CL implementation | | 54,293 |

Table SC3: Estimated annual cost savings following a nationwide CL implementation

|  |  |
| --- | --- |
| # of preventable indication-specific rehospitalizations following a nationwide CL implementation | 54,293 |
| Estimated cost savings (€) per indication-specific rehospitalization prevented | 6,978.23 |
| Estimated SHI savings following a nationwide CL implementation (€) | 378,869,572 |
